# Metabolic reconfiguration enables synthetic reductive metabolism in yeast

**DOI:** 10.1038/s42255-022-00654-1

**Published:** 2022-10-27

**Authors:** Tao Yu, Quanli Liu, Xiang Wang, Xiangjian Liu, Yun Chen, Jens Nielsen

**Affiliations:** 1 Department of Biology and Biological Engineering, Chalmers University of Technology, Gothenburg, Sweden; 2 Novo Nordisk Foundation Center for Biosustainability, Chalmers University of Technology, Gothenburg, Sweden; 3 Novo Nordisk Foundation Center for Biosustainability, Technical University of Denmark, Kongens Lyngby, Denmark; 4 Center for Synthetic Biochemistry, CAS Key Laboratory of Quantitative Engineering Biology, Shenzhen Institute of Synthetic Biology, Shenzhen Institutes of Advanced Technology, Chinese Academy of Sciences, Shenzhen, China; 5 BioInnovation Institute, Copenhagen, Denmark

**Keywords:** Metabolic engineering, Cellular microbiology, Cell biology, Biocatalysis, Metabolism

## Abstract

Cell proliferation requires the integration of catabolic processes to provide energy, redox power and biosynthetic precursors. Here we show how the combination of rational design, metabolic rewiring and recombinant expression enables the establishment of a decarboxylation cycle in the yeast cytoplasm. This metabolic cycle can support growth by supplying energy and increased provision of NADPH or NADH in the cytosol, which can support the production of highly reduced chemicals such as glycerol, succinate and free fatty acids. With this approach, free fatty acid yield reached 40% of theoretical yield, which is the highest yield reported for *Saccharomyces cerevisiae* to our knowledge. This study reports the implementation of a synthetic decarboxylation cycle in the yeast cytosol, and its application in achieving high yields of valuable chemicals in cell factories. Our study also shows that, despite extensive regulation of catabolism in yeast, it is possible to rewire the energy metabolism, illustrating the power of biodesign.

## Abstract (web summary)

In this study, Yu et al. engineer a decarboxylation cycle in the yeast cytoplasm that provides increased reductive power to enhance bioproduction of reduced chemicals.

## Main

Cellular function is the sum of a large number of coordinated chemical reactions, most clearly represented by catabolic processes where carbon and energy sources are converted to Gibbs free energy and the building blocks required for cellular proliferation^1,2^. In anabolic processes, building blocks are converted to macromolecules under the context of energy of consumption.

In these processes the cell also needs to process electron flows to overcome the stoichiometric constraints of chemical composition between the substrate and various macromolecules that form the biomass of the cell. For example, a typical yeast biomass chemical composition is CH_1.76_N_0.17_O_0.56_, which is slightly more reduced than glucose^3^, but some key components—for example, lipids—are reduced by far more than glucose. In biotechnology it is also desirable to produce highly reduced chemicals—for example, for use as biofuels. Thus, although it is necessary to enhance the capacity of reductive metabolism, it is very challenging to engineer this component of metabolism^4^.

Cells have evolved to possess multiple energy-producing pathways to maintain growth in response to varying conditions^5^. Because energy metabolism is very important to the cell, molecular regulators of energy metabolism have essential roles in stem cells, nuclear reprogramming and cancer cell fate^6^. In the heterotroph *Saccharomyces cerevisiae* there are two mechanisms by which ATP is produced: substrate-level and oxidative phosphorylation. Oxidative phosphorylation occurs during cellular respiration, and this process can utilize the oxidation of NADH/FADH_2_ to NAD^+^/FAD to generate ATP/GTP, whereas NADH/FADH_2_ is generated by the native reductive metabolism of cells, which involves both glycolysis and the tricarboxylic acid (TCA) cycle^7^. Through the TCA cycle and oxidative phosphorylation, the cell releases all the energy in glucose by generation of CO_2_.

Metabolic reactions must be highly coordinated to ensure a defined ratio between different building blocks and a defined ratio between energy generation and these building blocks^2^. We therefore wondered whether it is possible to create a synthetic energy system for the cell. What would happen if the cell was overcharged with NADH or NADPH? For heterotrophs, the TCA cycle is a series of chemical reactions used by all aerobic organisms to release stored energy from glucose by the oxidation of acetyl-CoA to CO_2_ and NADH, which further charges the electron transfer chain in the mitochondrial membrane for ATP generation^8^. The TCA cycle performs repeated decarboxylation to generate energy. Is this system universal, or can it be replaced? From an evolutionary perspective, every component or module of the cell, including energy metabolism, has been optimized for self-reproduction rather than production of a single chemical. Therefore, is it possible to create a synthetic energy system that can be used for optimized chemical production? Traditionally most strategies in metabolic engineering and synthetic biology, such as biosensor-based dynamic regulation, overexpression of pathways or deletion of specific enzymes, mainly focus on biosynthetic or anabolic processes^9,10^.

Here we report the reconstruction of a cytosolic, synthetic reductive metabolic pathway characterized by a repeated single decarboxylation reaction capable of supporting cell growth by replacement of the native TCA cycle in *S. cerevisiae*. More importantly, this synthetic pathway can be recruited to produce highly reduced chemicals. This synthetic reductive metabolism can be combined with the non-oxidative glycolysis (NOG) pathway^11^ for optimized acetyl-CoA-derived chemical production. This is also the first example, to our knowledge, of a synthetic energy system enabled by a rational biodesign that can support cell growth. Based on our findings we conclude that yeast metabolism, despite millions of years of evolution, is relatively plastic. Through rational design and extensive engineering it is feasible to redesign energy metabolism, the most basic part of life.

**Fig. 1:**
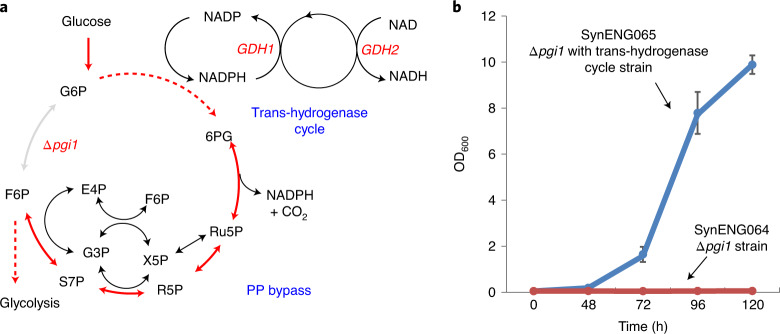
Detoxification of extra NADPH by the trans-hydrogenase cycle. **a**, Overview of the irreversible trans-hydrogenase cycle and PP bypass. *GDH1* and *GDH2* enable one irreversible trans-hydrogenase cycle. *GDH1*, NADP(^+^)-dependent glutamate dehydrogenase, synthesizes glutamate from ammonia and alpha-ketoglutarate. *GDH2*, NAD(^+^)-dependent glutamate dehydrogenase, degrades glutamate to ammonia and alpha-ketoglutarate. The oxidative and non-oxidative PPs form a metabolic bypass of *pgi1* deletion, which generate extra NADPH rather than glycolysis. **b**, Trans-hydrogenase cycle enabled the cell growth of *pgi1* deletion E1B strain SynENG064. SynENG065 was deleted by *pgi1* and overexpressed with *GDH1* and *GDH2* (trans-hydrogenase cycle). Source data

**Fig. 2:**
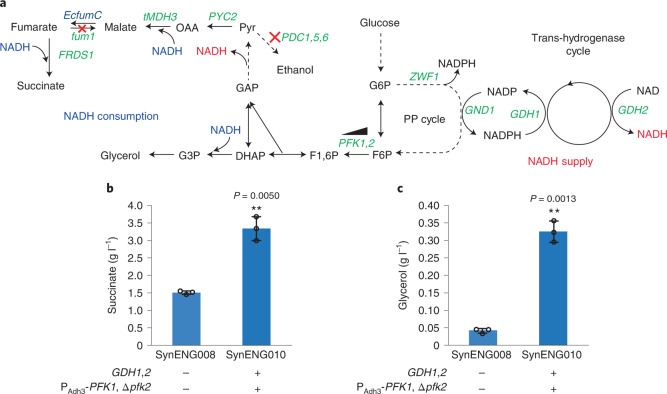
Synthetic energy system generates additional NADH for markedly reduced chemical production. **a**, Schematic illustration of PP cycle by downregulation of phosphofructokinase; genetic modifications in succinate overproduction strains SynENG008 and SynENG010 are also shown. For more detail see Extended Data Fig. 2. **b**,**c**, The PP cycle, together with the trans-hydrogenase cycle, increase the production of succinate (**b**) and glycerol (**c**). Strains were cultivated for 96 h in minimal medium and the final products were quantified by high-performance liquid chromatography. Statistical analysis was conducted using Student’s *t*-test (two-tailed, two-sample unequal variance, *P < 0.05, **P < 0.01, ***P < 0.001; sample size, *n* = 3). At least two independent measurements were performed for each experiment, and the mean ± s.d. of three biological replicates of a representative measurement is shown. All cells were grown as described in experimental procedures. Source data

**Fig. 3:**
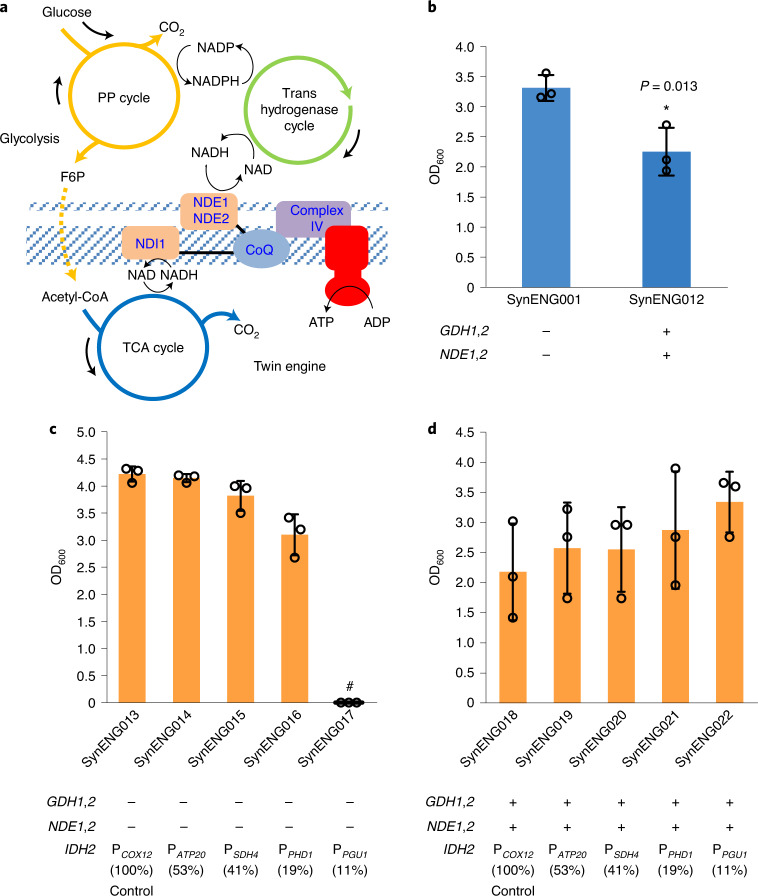
Synthetic energy system supports cell growth. **a**, Schematic illustration of twin-engine design. **b**, Excessive decarboxylation decreases cell growth. **c**, Insufficient decarboxylation decreases cell growth in the absence of the synthetic energy system. Fine-tuning of the TCA cycle was achieved by downregulation of *IDH2*. *IDH2* was expressed separately by a series of promoters from *COX12* (100%), *ATP20* (53%), *SDH4* (41%), *PHD1* (19%) and *PGU1* (11%). #, no surviving colony. **c**,**d**, PCOX12 refers to the promoter of COX12, same as PATP20, PSDH4, PPHD1 and PPGU1. **d**, Synthetic energy system replaces the TCA cycle to generate energy for cell growth. Native *IDH2* was expressed separately by a series of promoters from *COX12* (100%), *ATP20* (53%), *SDH4* (41%), *PHD1* (19%) and *PGU1* (11%). Strains were cultivated in shake flasks for 120 h at 200 r.p.m. and 30 °C with glucose feed beads corresponding to 10 g l^−1^ glucose in SD medium. Statistical analysis was conducted using Student’s *t*-test (two-tailed, two-sample unequal variance, *P < 0.05, **P < 0.01, ***P < 0.001; sample size, *n* = 3). At least two independent measurements were performed for each experiment, and the mean ± s.d. of three biological replicates of a representative measurement is shown. All cells were grown as described in experimental procedures. Source data

**Extended Data Fig. 1:**
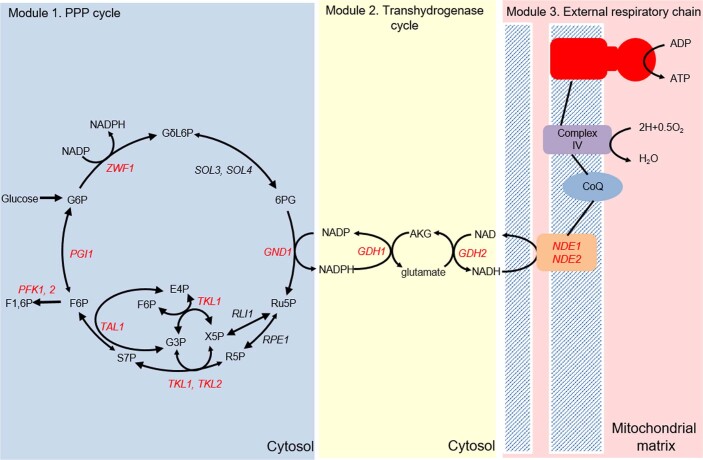
Schematic illustration of synthetic energy system. The synthetic energy system contains three modules. Firstly, the PP pathway was transformed into a PP cycle by downregulation of *PFK1/2*, which enabled the repeated decarboxylation. The H + was carried in the form of NADPH. Secondly, one transhydrogenase cycle was introduced, which transformed the NADPH into NADH. At last, the NADH in the cytosol was transfered to the respiratory chain in mitochondrial membrane to generate energy.

The synthetic energy system contains three modules: module 1, the pentose phosphate (PP) pathway cycle; module 2, the trans-hydrogenase cycle; and module 3, the external respiratory chain (Extended Data Fig. 1). In Fig. 1 we present the trans-hydrogenase reaction enabled by module 2. We then demonstrate the repeated decarboxylation and cytosol NADH supply function of module 1, which is reflected by highly reduced chemical overproduction (Fig. 2). Finally, we achieved the energy supply function by integration of all three modules (Fig. 3).

## Repeated decarboxylation

Decarboxylation is the basis for cellular reductive metabolism. For energy generation in many organisms the TCA cycle is central. In this pathway, repeated decarboxylation leads to the generation of NADH, which is subsequently reoxidized by the electron transport chain, eventually resulting in ATP production. The construction of a decarboxylation cycle is therefore the first step towards building a new energy system. For this purpose, we aimed to rewire the PP pathway into a PP cycle with repeated decarboxylation functions. The PP pathway can remove one CO_2_ from glucose-6-phosphate to generate ribulose-5-phosphate for cell growth. In this process, two NADPH + two H^+^ are generated per mole of carbon released—this is the oxidative PP phase. Then, a series of molecular rearrangements generate C3, C4, C6 and C7 from C5 sugars. Finally, three C5 molecules can form two fructose-6-phosphate molecules and one glyceraledhyde-3-phosphate molecule in the non-oxidative PP phase. Glyceraledhyde-3-phosphate and fructose-6-phosphate can be converted back by gluconeogenesis to glucose-6-phosphate, which can again enter the oxidative PP phase. Partial gluconeogenesis, the combined oxidative and non-oxidative components of PP, form a metabolic cycle^12^ that we here term the PP cycle (Fig. 1a and Extended Data Fig. 1). In this pathway, a total of 12 moles of NADPH per mole of glucose are generated. NADPH is primarily used as a reductant in biosynthesis pathways whereas NADH is mainly used in energy metabolism, where it enters the respiratory chain as an electron donor for energy generation. There are three potential design options to transforming NADPH to NADH: (1) overexpression of the soluble trans-hydrogenase UdhA from *Escherichia coli*^13,14^; (2) overexpression of glyceraldehyde-3-phosphate dehydrogenase (GAPDH), which accepts both NADP^+^ and NAD^+^^15^; and (3) overexpression of NAD^+^-dependent glutamate dehydrogenase (GDH2)^16,17^. Here, we prefer the irreversible trans-hydrogenase cycle by overexpression of native genes *GDH1* and *GDH2*. Gdh1p, an NADP^+^-dependent glutamate dehydrogenase, synthesizes glutamate from ammonia and alpha-ketoglutarate; Gdh2p, an NAD^+^-dependent glutamate dehydrogenase, degrades glutamate to ammonia and alpha-ketoglutarate. Combined, these enzymes form a cycle between glutamate from ammonia and alpha-ketoglutarate. In this cycle, one NADPH was irreversibly transferred into one NADH (Figs. 1a and 2a and Extended Data Fig. 1).

To generate sufficient energy for cell growth, the PP cycle should be able to facilitate a relatively high carbon flux. To test the capacity for carbon flux in oxidative and non-oxidative PPP, we deleted the *pgi1* gene to create a ‘PP bypass’ for glucose metabolism. As shown in Fig. 1b, without the *pgi1* gene the strain E1B (SynENG001), an evolved *pdc*- strain from wild-type yeast^18^, cannot grow on glucose as the sole carbon source, probably due to excess NADPH generation (SynENG064). However, in the context of a functional trans-hydrogenase cycle, the cells successfully grew to optical density OD_600_ = 10 after 5 days of cultivation (SynENG065). By blocking glycolysis at the phosphoglucose–isomerase node, all glucose should be forced through the PP pathway. The E1B strain is an evolved *pdc*-negative strain that cannot produce ethanol. With the abolition of fermentation (mainly an energy supply from substrate-level phosphorylation), oxidative phosphorylation is the major energy source in the cell. This strain has the ability to process NADH in the cytosol without the reduction of pyruvate to ethanol, even in high-glucose medium^18^. In wild-type *S. cerevisiae*, *pgi1* null mutants cannot grow on glucose^16,17^. Overproduction of NAD^+^‐dependent glutamate dehydrogenase (GDH2) can suppress the phenotype of cells lacking the phosphoglucose isomerase *PGI1*. Together with NADPH‐dependent glutamate dehydrogenase (GDH1), a cyclic trans-hydrogenase system, consumption of NADPH and generation of NADH occurred^19^. This result is consistent with previous studies^19^. The defective growth of the Δ*pgi1* strain on glucose can also be restored by expression of *E. coli* trans-hydrogenase udhA or NADP-GAPDH. However, not all *S. cerevisiae* strains lacking PGI1 can be rescued by the expression of trans-hydrogenase^14^. Based on our results, we propose that this growth defect may also be due to the lack of a functional respiratory system capable of oxidizing additional NADH from NADPH. Together, these results demonstrate that the growth limitation of the Δ*pgi1* strain may be due to the lack of a mechanism for reoxidation of surplus NADPH.

Our results clearly demonstrate that the oxidative and non-oxidative PP pathways, and the activity of the trans-hydrogenase cycle, can support cell growth, thus providing a promising entry point for the construction of a synthetic energy system.

## Highly reduced chemical production

Products and cell chassis naturally suffer stoichiometric constraints from available substrates, leading to biosynthetic imbalance and suboptimal product yield. When highly reduced chemicals are produced from glucose, the provision of reducing power in the cytoplasm of eukaryotes is generally rate limiting due to the compartmentation of NADH metabolism.

In the TCA cycle, two molecules of CO_2_ are released and the electrons are preserved as NADH. Compared with the TCA cycle, the synthetic PP cycle, which is localized in the cytoplasm, recursively oxidizes carbons from glucose and releases one molecule of CO_2_ while preserving the electrons as NADPH. We propose that obligatory NADPH synthesis through the PP cycle can further stimulate carbon reduction. When combined with the trans-hydrogenase cycle, obligatory NADH synthesis can be achieved through this recursive use of the oxidative PP pathway. This design should enable simple fine-tuning of NADH and NADPH supply in the cytoplasm of eukaryotes.

**Extended Data Fig. 2:**
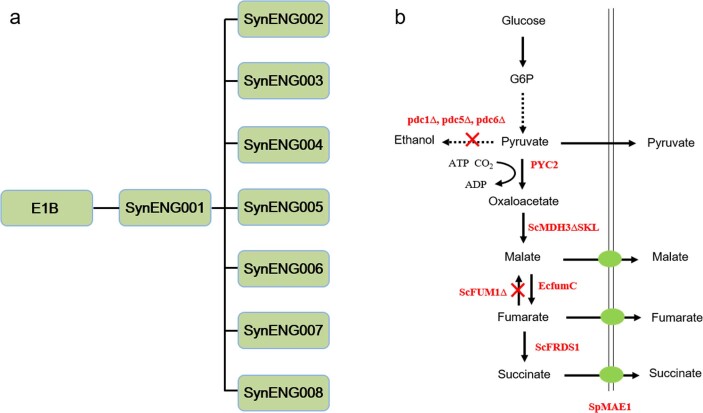
The genetic modifications in the strains for Fig. 2. **a**, Flowchart of the related yeast strain in this study. More detail information was shown in Supplementary Table 2. **b**, The construction of succinate acid production strain. The E1B strain was used as the background strain, which is one evolved *pdc* negative strain. The native *PYC2* and *FRDS1* was overexpressed. The native *MDH3* was relocalized into cytosol by deletion the C-terminal signal. One *FumC* gene from E.coli was introduced into the strain, and the native *FUM1* was deleted.

Succinic acid, a four-carbon dicarboxylic acid, has been traditionally used as a surfactant and additive in the agriculture and food industries^20^. The reductive succinate pathway has high yield because it fixes one carbon dioxide^20^. In *S. cerevisiae*, NADH is produced by conversion of glucose to pyruvate in the cytosol by GAPDH. The additional reducing power of NADH is required to produce succinate from pyruvate by this pathway. We simultaneously overexpressed pyruvate carboxylase (PYC2), retargeted the peroxisomal malate dehydrogenase (ScMDH3△SKL) into the cytosol, expressed a fumarase from *E. coli* (EcfumC), expressed the endogenous fumarate reductase (ScFRD1)^21^ and overexpressed the *Schizosaccharomyces pombe* malate transporter gene Sp*MAE1* in a previously engineered E1B strain (SynENG001) that is unable to undergo alcoholic fermentation (Fig. 2a and Extended Data Fig. 2). To force carbon flux into the synthetic PP cycle, phosphofructokinase was downregulated by the deletion of *PFK2* and downregulation of *PFK1*. As expected, combining the synthetic PP cycle and trans-hydrogenase cycle resulted in a marked increase in the titre of succinate, to approximately 3.3 g l^–1^ (Fig. 2b). We also observed glycerol production in response to increasing NADH (Fig. 2c). Glycerol production in *S. cerevisiae* is a consequence of an imbalance in cytosolic NADH^3^. These results clearly demonstrate that additional NADH was successfully generated.

## Synthetic reductive metabolism enables an energy system supporting cell growth

For the native energy system, NADH can be repeatedly generated in mitochondria through the TCA cycle. One single-subunit NADH-ubiquinone oxidoreductase couples the oxidation of intramitochondrial NADH to the respiratory chain. The enzyme Ndi1, which is referred to as ‘internal NADH dehydrogenase’, catalyses the transfer of two electrons from intramitochondrial NADH to ubiquinone in the respiratory chain^22,23^. The electron transport chain comprises complexes I–IV, which extract energy from the electron flow and use it to expel protons across the inner mitochondrial membrane. With the synthetic PP and trans-hydrogenase cycles we successfully achieved repeated decarboxylation in the cytosol. To capture energy from the generated NADH in the cytosol we overexpressed the two external NADH dehydrogenase isoenzymes, NDE1 and NDE2 (refs. ^3,24^), which can fill the gap between cytosolic NADH and the electron transport chain in the mitochondrial membrane for ATP generation (Fig. 3a and Extended Data Fig. 1).

To test this hypothesis, the trans-hydrogenase cycle and mitochondrial external NADH dehydrogenase isoenzymes were overexpressed in the evolved *pdc*-negative, E1B-derived strain (SynENG001). Non-synonymous mutations in *MTH1* alleviated glucose repression in the E1B strain by repressing the expression of several hexose transporter genes, which restrict glucose transport and provide the strain with functional oxidative respiration based on the TCA cycle with glucose. With the abolition of fermentation (mainly energy supply from substrate-level phosphorylation), oxidative phosphorylation is the major energy source in this strain. Using this synthetic energy design, the cell growth of SynENG012 was even weaker than that of the control strain (Fig. 3b). We propose that this finding indicates that excessive decarboxylation/NADH generation or imbalance of NADH and NADPH is toxic to cells. To further validate this hypothesis, the native TCA cycle was downregulated by either replacing the promoter of *IDH2* (subunit 2 of mitochondrial NAD(^+^)-dependent isocitrate dehydrogenase) with weaker alternatives with or without the second-engine design (synthetic energy system)^25^. Without the synthetic energy system, as the promoter weakened cell growth weakened and no surviving colonies were observed on the plate (Fig. 3c). We propose that this cell growth defect was due to lack of energy. However, in the context of the synthetic energy system, as *IDH2* expression became weaker cell growth improved (Fig. 3d; Student’s *t*-test was conducted between strains SynENG017 and SynENG022, *P* = 0.11). Comparing strains SynENG017 and SynENG022 using the same *PGU1* promoter for *IDH2*, we observed that the synthetic energy system can replace the TCA cycle to supply energy for enhanced cell growth.

## Acceleration of lipogenesis by synthetic reductive metabolism

**Fig. 4:**
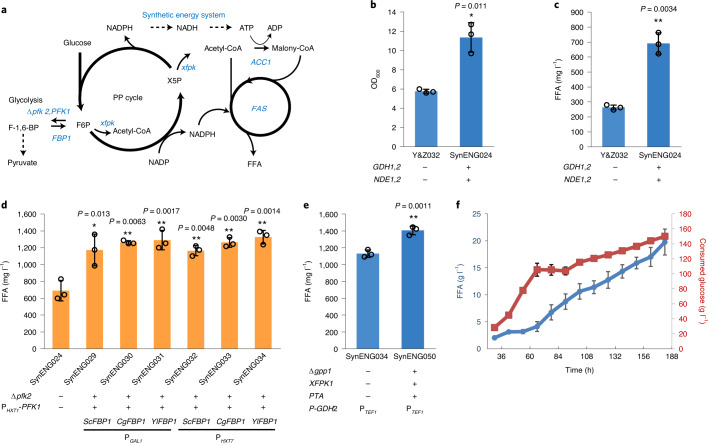
Synthetic energy system enhances FFA production. **a**, Schematic illustration of the twin engine for FFA production. **b**, The synthetic energy system can replace the TCA cycle to support cell growth. **c**, The synthetic energy system can replace the TCA cycle to support FFA production. **d**, Increased decarboxylation benefits FFA production. **e**, The NOG pathway, in concert with the synthetic energy system, increased FFA production. **a**–**e**, Statistical analysis was conducted using Student’s *t*-test (two-tailed, two-sample unequal variance, *P < 0.05, **P < 0.01, ***P < 0.001; sample size, *n* = 3). At least two independent measurements were performed for each experiment, and the mean ± s.d. of three biological replicates of a representative measurement is shown. All cells were grown as described in experimental procedures. **f**, Fed-batch fermentation of strain SynENG058 under glucose-limited and nitrogen-restricted conditions. The plots include both batch and fed-batch data. Centre and error bars represent mean ± s.e.m. (*n* = 3 biologically independent samples). Source data

**Extended Data Fig. 3:**
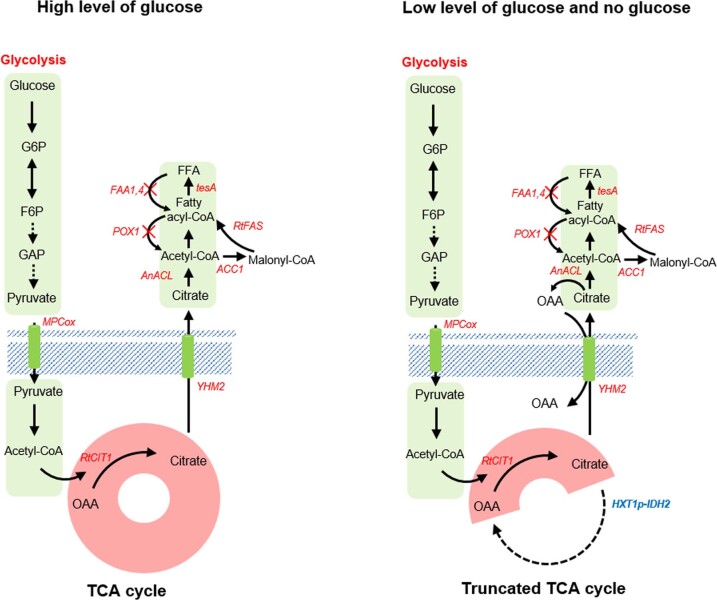
The genetic modifications in the strains Y&Z032. The *IDH2* (Subunit 2 of mitochondrial NAD( + )-dependent isocitrate dehydrogenase) gene in Y&Z032 is expressed by the *HXT1* promoter, which is induced at high and suppressed at low glucose concentrations. Other modifications were also shown in Figure.

In oleaginous fungi, the initiation of lipid overproduction is triggered by impaired activity of mitochondrial NAD(^+^)-dependent isocitrate dehydrogenase Idhp^26^, resulting in citrate export from mitochondria to the cytosol. To simulate this effect and channel additional carbon flux into the prduction of free fatty acids (FFAs), in our previous study^25^ we abolished Idhp activity by deletion of *IDH1* (subunit 1 of mitochondrial NAD(^+^)-dependent isocitrate dehydrogenase) and/or *IDP1* (mitochondrial NADP-specific isocitrate dehydrogenase). However, deletion of *IDH1* (strain Y&Z047) resulted in a much lower FFA titre with reduced biomass yield, and the double *IDH1*–*IDP1* deletion was lethal. To dynamically control the expression of isocitrate dehydrogenase, the *IDH2* promoter was exchanged for the *HXT1* promoter, which is induced by high glucose concentrations and suppressed by low glucose concentrations, to create the strain Y&Z032. In strain Y&Z032, FFA biosynthesis and the ATP-citrate lyase-based acetyl-CoA pathway were systematically optimized. Further details are shown in Extended Data Fig. 3. We evaluated strain performance in flasks using glucose slow-release feed beads, which can simulate glucose-limited fermentation to avoid the Crabtree effect. We then discovered that both cell growth and FFA production of Y&Z032 were reduced. We propose that the lower FFA titre with reduced biomass yield was caused by a shortage of energy supply due to either a dynamic TCA cycle or dynamic *IDH2* expression. To validate this hypothesis, we introduced the synthetic energy system, the trans-hydrogenase cycle and mitochondrial external NADH dehydrogenase isoenzymes into strain Y&Z032. Biomass yield was then improved by approximately 100% (Fig. 4b) but, more interestingly, the FFA titre increased by approximately 200% (Fig. 4c) because FFA production was partially coupled with cell growth. These results further demonstrate that the synthetic energy system can support cell growth and the production of highly reduced chemicals.

**Extended Data Fig. 4:**
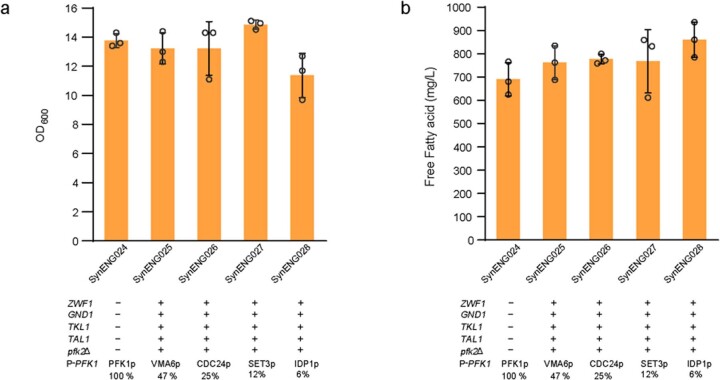
Synthetic energy system support FFAs production. Increased decarboxylation benifit FFAs production. Fine-tune of glycolysis was achieved by down regulation of phosphofructokinase PFK1 and the deletion of PFK2. The native *PFK1* promoter (100%) was replaced by weaker promoter of *VMA6p*(47%), *CDC24*(25%), *SET3*(12%), *IDP1*(6%), separately. The cell growth and fatty acid level were shown in figure **a** and figure **b**. Statistical analysis was conducted using a Student’s *t*-test (one-tailed; two-sample unequal variance; *P < 0.05, **P < 0.01, ***P < 0.001; sample size, *n* = 3). At least two independent measurements were performed for each experiment and the mean±s.d. of three biological replicates of a representative measurement is shown. All cells were grown as described in experimental procedures. Source data

We then tried to enhance the PP cycle by overexpression of key metabolic genes involved in the following pathways: (1) *ZWF1*, encoding glucose-6-phosphate dehydrogenase, which catalyses the irreversible and rate-limiting first step of the PP pathway and is predominantly responsible for NADPH regeneration from NADP^+^; (2) *GND1*, encoding the major phosphogluconate dehydrogenase that catalyses the second oxidative reduction of NADP^+^ to NADPH; and (3) *TKL1* and *TAL1*, encoding transketolase and transaldolase, respectively, both of which are part of the non-oxidative branch of the PP cycle. Combined with *PFK2* deletion and various promoters of *PFK1*, overexpression of these enzymes substantially improved FFA production as shown in Extended Data Fig. 4. Based on these data, NADPH and/or ATP are probably still the rate-limiting steps for FFA biosynthesis.

**Extended Data Fig. 5:**
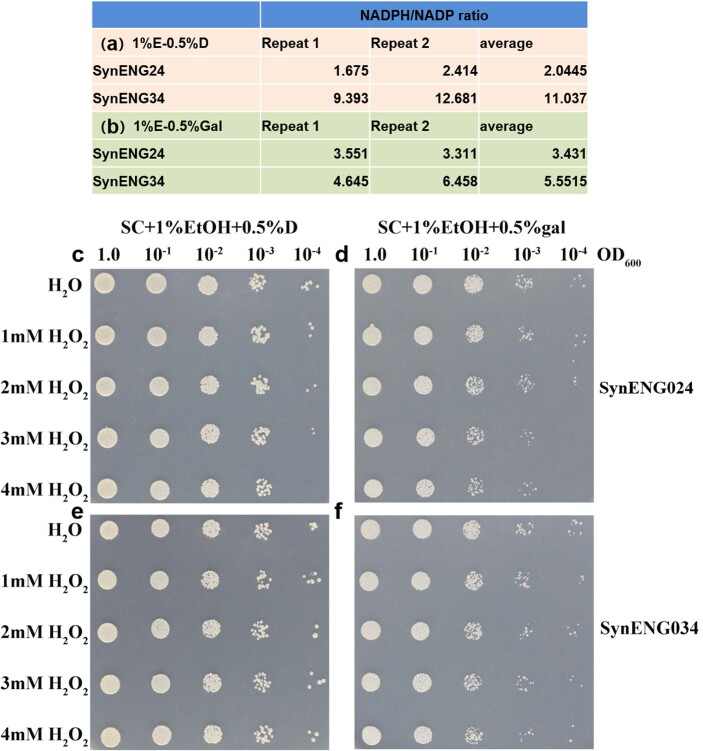
Synthetic reductive metabolism increase the NADPH/NADP + ratios. **a**, Measurements of intracellular ratios of NADPH/NADP + in SynENG024 and SynENG034 grown in 1%+0.5% glucose. **b**, Measurements of intracellular ratios of NADPH/NADP + in SynENG024 and SynENG034 grown in 1%+0.5% galactose. Results show average of three independent experiments, Error bars denote standard deviations. **c**, Growth of yeast cells SynENG024 in presence of stressing agent (H_2_O_2_) on SD/-Ura basal medium with 1% ethanol and 0.5% glucose. **d**, Growth of yeast cells SynENG034 in presence of stressing agent (H_2_O_2_) on SD/-Ura basal medium with 1% ethanol and 0.5% glucose. **e**, Growth of yeast cells SynENG024 in presence of stressing agent (H_2_O_2_) on SD/-Ura basal medium with 1% ethanol and 0.5% galactose. **f**, Growth of yeast cells SynENG034 in presence of stressing agent (H_2_O_2_) on SD/-Ura basal medium with 1% ethanol and 0.5% galactose. Related to Fig. 4. Source data

It has previously been shown that overexpression of the gluconeogenic enzyme fructose-1,6-bisphosphatase could increase NADPH supply in *Corynebacterium glutamicum* via the redirection of glycolytic flux to the PP pathway^25^. In addition to overexpression of the fructose-1,6-bisphosphatase-coding gene, *FBP1*, we also downregulated the activity of phosphofructokinase by simultaneously deleting gene *PFK2* and implementing dynamic control over *PFK1* gene expression (Fig. 4a). Specifically, we tested three different *FBP1* homologues from *S. cerevisiae* (*ScFBP1*), *C. glutamicum* (*CgFBP1*) and *Yarrowia lipolytica* (*YlFBP1*). To avoid the potentially futile cycle between *PFK1* and *FBP1*, different carbon source-responsive promoters were used, as shown in Fig. 4d. *PFK1* was expressed by the *HXT1* promoter, which is induced at high glucose concentrations and suppressed at low glucose concentrations. The expression of different background *FBP1* genes was driven by *GAL1p*, which is induced by the presence of galactose, or by *HXT7p*, which is suppressed at high glucose concentrations and induced at low glucose concentrations, or by the addition of galactose. As shown in Fig. 4d, the FFA titre was further improved (by approximately 90%) by shunting extra carbon flux into the PP cycle of the synthetic energy system by the optimal strains SynENG031 and SynENG034. Strain SynENG034 showed a higher NADPH:NADP^+^ ratio than SynENG024 (Extended Data Fig. 5b). NADPH is a molecule that is essential for free radical detoxification and stabilization of cell redox state, because it provides reducing capacity for the circulation of antioxidant enzymes. SynENG034 also shows greater tolerance to oxidants such as H_2_O_2_ (Extended Data Fig. 5d).

**Extended Data Fig. 6:**
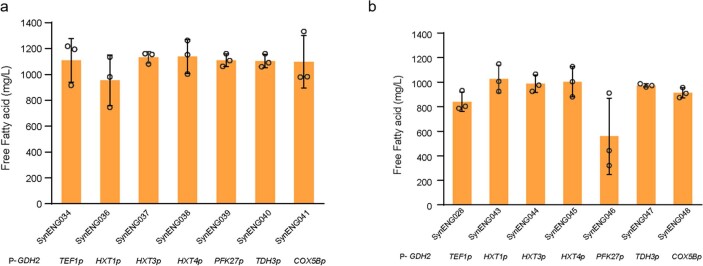
Dynamic regulation of the transhydrogenase cycle. **a**, Dynamic regulation of the transhydrogenase cycle have no effect on FFAs production with high NADPH supply. Dynamic regulation of the transhydrogenase cycle was achieved by fine-tunning of GDH2 expression. Canbon source dependent promoters, such as *HXT1, HXT3, HXT4, PFK27 and COX5B*, were used. These promoters were induced by high levels of glucose and repressed by low levels of glucose. *TDH3* promoter was used as a second control. The background strain SynENG034 have high level of NADPH supply as shown in Fig. 4d. **b**, Dynamic regulation of the transhydrogenase cycle have no effect on FFAs production with low NADPH supply. Dynamic regulation of the transhydrogenase cycle was achieved by fine-tunning of GDH2 expression. Canbon source dependent promoters, such as *HXT1, HXT3, HXT4, PFK27 and COX5B*, were used. These promoters were induced by high levels of glucose and repressed by low levels of glucose. *TDH3* promoter was used as a second control. The background strain SynENG034 have high level of NADPH supply as shown in Extended Data Fig. 3. Statistical analysis was conducted using a Student’s *t*-test (one-tailed; two-sample unequal variance; *P < 0.05, **P < 0.01, ***P < 0.001; sample size, *n* = 3). At least two independent measurements were performed for each experiment and the mean±s.d. of three biological replicates of a representative measurement is shown. All cells were grown as described in experimental procedures. Source data

Lipogenesis is a typical reductive metabolic process that produces fatty acids from glucose. It requires a balanced supply of NADPH, ATP and precursor acetyl-CoA at a 2:1:1 ratio. We demonstrated that the ratio between cofactor (NADPH, ATP) and precursor acetyl-CoA could be fine-tuned by optimization of the expression level of FBP1 and PFK1 (Fig. 4d). Moreover, based on our design and by fine-tuning of *GDH2* and the trans-hydrogenase cycle, we could adjust the ratio between NADPH and ATP to further improve FFA titre. To test this hypothesis in strain SynENG034, the *TEF1* promoter for *GDH2* was replaced by a series of dynamic promoters, including the promoters from *HXT1*, *HXT3*, *HXT4*, *PFK27* and *COX5B*, which were induced by high levels of glucose and repressed by low levels^27^. However, we did not observe any substantial improvement in FFA titre (Extended Data Fig. 6a). We then evaluated this strategy in strain SynENG028. In this strain, the ratio between cofactor and precursor acetyl-CoA was lower than that in SynENG034 (Extended Data Fig. 4 and Fig. 4d), which resulted in no increase in FFA titre (Extended Data Fig. 6b). We expect that the reason for this finding may be that the yeast cell controls the ratio between NADPH and ATP production such that it matches the need for fatty acid production based on cell growth. Given that we decreased the conversion of F6P to F1,6P, carbon flux to the precursor acetyl-CoA derived from cytosol acetate or mitochondrial citrate may have limited FFA biosynthesis. We therefore implemented a heterologous phosphoketolase pathway^28^, consisting of a phosphoketolase from *Bifidobacterium breve* (*Bbxfpk*) and a phosphotransacetylase from *Clostridium kluyveri* (*Ckpta*) with the deletion of *GPP1* (refs. ^29,30^), to enhance the supply of acetyl-CoA. Phosphoketalose is able to split F6P and X5P into acetyl-phosphate (Fig. 4a). By doing so, part of the carbon flux from the PP cycle could be directly diverted toward generation of acetyl-CoA. As expected the FFA titre was further improved, by approximately 30% (Fig. 4e). This system serves as a good illustration of the challenge involved in engineering cell metabolism. Specifically, it is necessary to combine several different strategies for the best results because there is rarely a single bottleneck associated with overproduction of a given metabolite.

## Fed-batch fermentation of engineered strains

**Extended Data Fig. 7:**
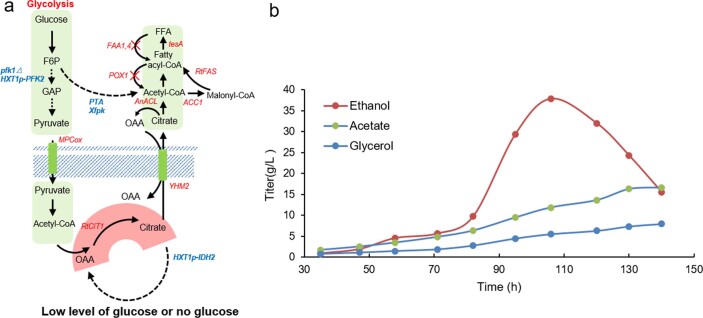
Fed-batch fermentation of strains SynENG050 under glucose limited and nitrogen restriction conditions. **a**, The genetic modifications in the strains SynENG050. **b**, Time courses of ethanol (red symbols), Acetate (green symbols) and Glycerol (light blue symbols) during the fermentation are shown. Source data

**Extended Data Fig. 8:**
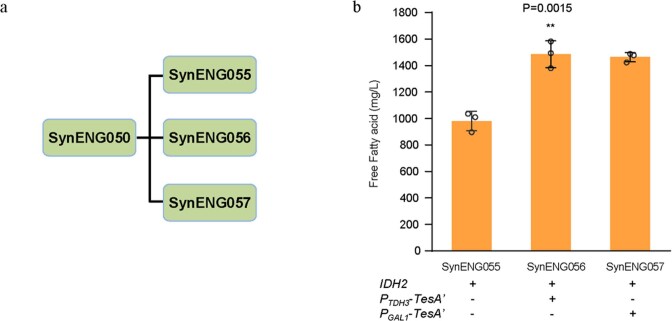
Optimization of TCA cycle and TesA’ expression for FFA production. **a**, Flowchart of the related yeast strain in this study. More detail information was shown in Supplementary Table 2. **b**, The FFAs production level. The strains were cultivated in shake flasks for 80 h at 200 r.p.m., 30 °C with glucose feed beads corresponding to 30 g/L glucose. Glucose feed beads release glucose slowly and therefore prevent ethanol production. Statistical analysis was performed using one-tailed Student’s *t*-test (*p < 0.05, **p < 0.01, ***p < 0.001). All data represent the mean ± SD of biological triplicates. Source data

**Extended Data Fig. 9:**
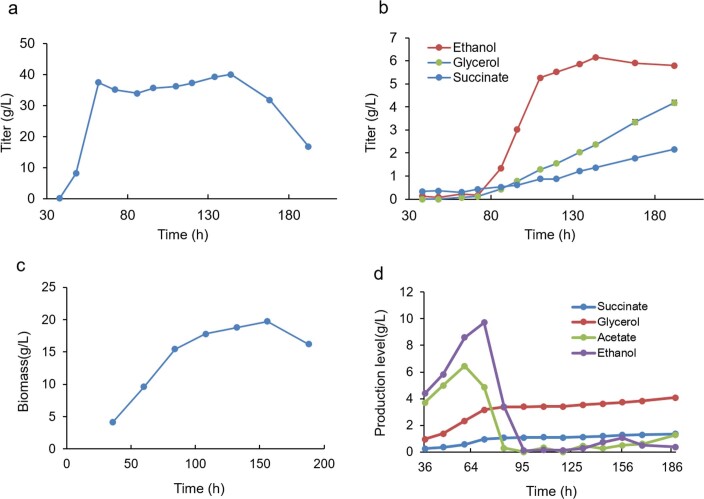
Fed-batch fermentation under glucose limited and nitrogen restriction conditions. **a**, Time courses of residual glucose during the fermentation of strains SynENG056. **b**, Time courses of ethanol (red symbols), glycerol (green symbols) and succinate(light blue symbols) during the fermentation of strains SynENG056 are shown. **c**, Time courses of dry cell weight during the fermentation of strains SynENG058. **d**, Time courses of succinate (blue symbols), glycerol (red symbols), acetate (green symbols) and ethanol (purple symbols) during the fermentation of strains SynENG058 are shown. Source data

**Extended Data Fig. 10:**
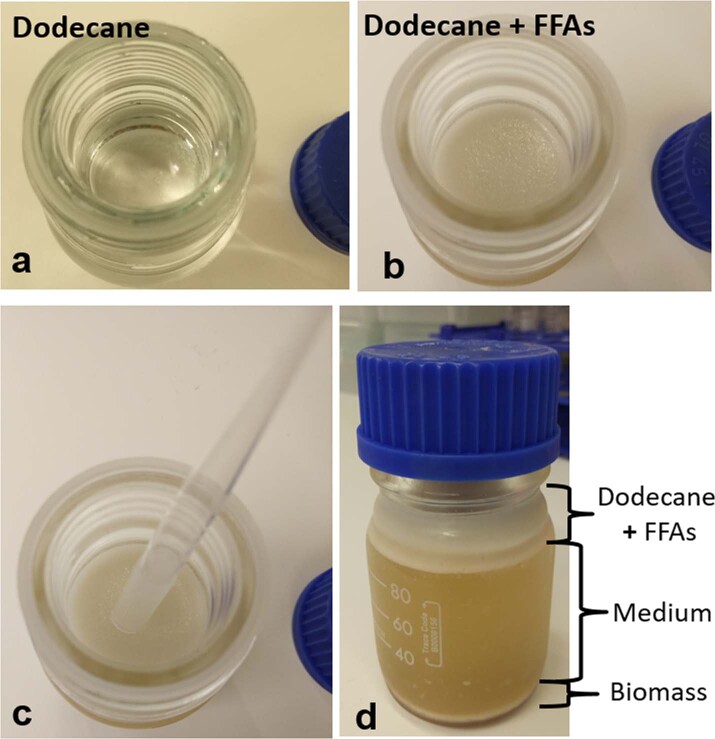
Dodecane extraction. **a**, Pure dodecane. **b**, Dodecane with FFAs after fermentation. **c**, Dodecane with FFAs after fermentation can support the weight of one 1 ml pipet tips. **d**, Dodecane layers after fermentations. (One bottle of about 120 ml medium after fermentation was stored in 4 °C for 12 h.). Source data

Shake-flask cultivation is useful for the construction and optimization of microbial cell factories; however, they generally fail to uncover the industrial potential of strains as a result of limited cultivation control. We therefore characterized the optimal FFA producer, SynENG050, in glucose-limited and nitrogen-restricted fed-batch cultivation. However, because the strain accumulated a high level of ethanol due to the use of the *HXT1* promoter for gene *IDH2*, the expression of this key gene of the TCA cycle would be induced by a high level of glucose and repressed by a low level. In other words, once excessive glucose levels are attained, *S. cerevisiae* will accumulate ethanol by fermentation due to the Crabtree effect. However, when we reduced the rate of glucose feeding the *HXT1* promoter was repressed. Without *IDH2*, cells with a damaged TCA cycle were not able to fully consume the overproduced ethanol. Moreover, as shown in Extended Data Fig. 7, the accumulation of acetate corroborated the cell growth defect of strain SynENG034 with ethanol or acetate as the carbon source. To solve this problem and generate a more robust strain, we instead expressed *IDH2* under its native promoter to obtain a fully functional TCA cycle in SynENG050, resulting in SynENG055. However, the titre of SynENG055 was reduced to 980 from 1,405 mg l^–1^ (strain SynENG050). We hypothesize that an overly strong TCA cycle competes for the carbon flow of fatty acid synthesis. To further strengthen the carbon flow to FFA synthesis, another copy of thioesterase TesA’ was introduced, resulting in an increased FFA titre of approximately 40% (strain SynENG056; Extended Data Fig. 8). The titre of SynENG056 returned to 1,485 mg l^–1^. We then characterized the optimal strain, SynENG056, in fed-batch cultivation. However, this strain accumulated a large amount of glucose (Extended Data Fig. 9a). Correspondingly, the cell also produced large amounts of glycerol and succinate (Extended Data Fig. 9b), the biosynthesis of which requires an abundant NADH supply in the cytosol. These data show that glycolysis flux was effectively reduced at the point of F6P and channelled into the PP cycle, implying that our synthetic energy system is functional under fermenter conditions. We propose that the accumulation of glucose and competing by-products is due to weakness of the FFA pathway or/and FAA export, which could consume more NADPH and thus reduce NADH generation. To validate this hypothesis we overexpressed whole-pathway genes including *FAS1*, *FAS2*, *Xfpk*, *PTA*, *TesA’* and *ACC1*^S659A, S1157A^, which resulted in strain SynENG058. We evaluated this strain in a fermenter with a dodecane layer, which could further increase the export of FFAs. With dodecane extraction, SynENG058 produced almost 20 g l^–1^ FFAs (Fig. 4f) with a yield of approximately 0.134 g FFA g^–1^ glucose, which corresponds to 40% of the maximum theoretical yield (Extended Data Fig. 9c,d). Following fermentation the dodecane layer with FFAs turned solid at 4 °C, as shown in Extended Data Fig. 10. This result indicated the high concentration of FFAs in the dodecane layer.

## Discussion

To ensure proper provision of key building-block metabolites—energy in the form of ATP and biosynthetic-reducing power in the form of NADH/NADPH, which should be synthesized in proper stoichiometric ratios—cells have evolved to incorporate several pathways operating in a highly coordinated fashion. One of the greatest biotechnological challenges involves changing these stoichiometric ratios based on metabolic network rigidity^31^. In addition, different substrates have different degrees of constraint, which will result in some metabolic intermediates or ATP to be out of balance and wasted.

To address this challenge, we present the potential of a synthetic reductive metabolic pathway as a generalizable method for cell energy generation and the ability of the cell to produce reduced bioproducts. One of the key successes of our synthetic reductive metabolism includes improving the efficiency of the synthesis of more reduced compounds from input substrates of variable oxidation state. From glucose (C_6_H_12_O_6_) to biomass (a typical yeast biomass chemical composition is CH_1.76_N_0.17_O_0.56_, which is slightly more reduced than glucose)^3^, cell metabolism was optimized for chemical reactions by one stoichiometric constraint on available carbons, energy and redox cofactors, resulting in biosynthetic imbalance and suboptimal product yield of more reduced chemicals^4^. Our design can alter these stoichiometries, with the potential to alleviate such stoichiometric constraints in reductive metabolism by fine-tuning ratios among carbon (precursor), energy (ATP) and cofactors (NADH and NADPH). We can overcome the limitation of ATP- and NADPH-dependent biological carbon reduction.

By rewiring the energy metabolism we created an artificial energy system. This system relies on the production of cytosolic NADPH by the oxidative component of the PP pathway that is designed to operate in a cyclic fashion with repeated decarboxylation. We demonstrated that this synthetic repeated decarboxylation cycle can replace the TCA cycle for energy generation. This design would also have the potential to free the TCA cycle from its exclusive role in energy generation, and it could be used in Crabtree-negative or -positive strains with glucose-limited, fed-batch fermentation. A completely disconnected TCA cycle may also be considered in regard to overproduction of TCA intermediate derivative chemicals, especially when such production consumes a large amount of ATP.

*Saccharomyces cerevisiae* metabolism has been systematically optimized for FFA production in the past 10 years^32^. Using the synthetic reductive metabolism developed here we could further improve fatty acid production with our engineered strain, which, as far as we are aware, reached the maximum yield for FFA production in *S. cerevisiae* reported to date. This artificial synthetic energy system can supply extra NADH, NADPH and ATP to the cell. However, in regard to FFA overproduction, there are at least four key points: (1) precursor acetyl-CoA supply; (2) cofactor NADPH and ATP supply; (3) deregulation of the pathway through various factors, including FAS1, FAS2 and ACC1; and (4) secretion—that is, dodecane extraction used in our study. The productivity of FFAs was relatively low, at 0.11 g l^–1^ h^–1^. This low value is attributed to the notion that the feeding speed of glucose has to be low (Fig. 4f), which indicates low efficiency due to the low fitness of the synthetic energy system (Fig. 3d). With further improvements in fitness, we have confidence that the production yield and productivity of FFAs by *S. cerevisiae* could be further enhanced. Heterogenetic microbial production of FFAs by resting *S. cerevisiae* cells represents the next milestone in this field. This goal would require complete uncoupling of fatty acid biosynthesis from cell growth and a deep understanding of cell lipid metabolism. Another direction for heterogenetic FFA production would be to reduce material costs and minimize the environmental footprint in a carbon-neutral or -negative manner, such as using chemicals from semiphotosynthetic or electrochemical carbon fixation^33^.

In summary, our results demonstrate the establishment of a synthetic reductive metabolism/energy system that enables additional NADH supply in the cytosol. This pathway can support cell growth by replacement of the TCA cycle. Furthermore, this synthetic reductive metabolism improved FFA production in *S. cerevisiae*. The associated energy metabolic reprogramming demonstrates that *S. cerevisiae* metabolism is highly complex yet remarkably plastic. The organizational forms of cells are very diverse and flexible. Engineering of organisms enables studies of the general organizing principle of life.

## Methods

### Strains, plasmids and cultivation conditions

All codon-optimized heterologous genes were synthesized (Genscript) and are listed in Supplementary Table 1, and all strains used are listed in Supplementary Table 2. Either the DNA assembler method or the Gibson assembly cloning kit (New England Biolabs) was used for plasmid construction. All strains were derived from E1B (*MATa*, *ura3-52*, *his3*Δ1, *pdc1*Δ, *pdc5*Δ, *pdc6*Δ) or IMX581 (*MATa ura3-52 can1*Δ::*cas9-natNT2 TRP1 LEU2 HIS3*). A LiAc/SS carrier DNA/PEG method was used for yeast transformation. CRISPR–Cas9-mediated genome engineering was used for other chromosome-based gene knockout, promoter replacement and gene integration, as described previously^34^. YPD medium (10 g l^−1^ yeast extract, 20 g l^−1^ peptone and 20 g l^−1^ glucose; all from Merck Millipore) was used for regular cultures of yeast strains. YPD + G418 medium containing 200 mg l^−1^ G418 (Formedium) was used for the selection of transformants with a *kanMX* cassette. CSM-Ura medium containing 20 g l^−1^ glucose, 6.7 g l^−1^ yeast nitrogen base without amino acids (YNB; Formedium) and 0.77 g l^−1^ complete supplement mixture without uracil (CSM-Ura; Formedium) was used for the selection of transformants prototrophic to uracil. CSM + 5-FOA medium, containing 6.7 g l^−1^ YNB, 0.79 g l^−1^ complete supplement mixture (CSM; Formedium) and 0.8 g l^−1^ 5-fluoroorotic acid (5-FOA; Sigma–Aldrich), was used for recycling of the *URA3* marker. Next, 20 g l^−1^ agar (Merck Millipore) was added to prepare solid media. When needed, 100 mg l^−1^ histidine and/or uracil was added to the medium. If not specified, Delft medium^35^ with 7.5 g l^−1^ (NH_4_)_2_SO_4_ as nitrogen source was used, with 3 ml of dodecane added to 20 ml of liquid medium for in situ extraction.

### Shake-flask cultivation

Shake-flask batch fermentations for the production of FFAs were carried out in minimal medium^35^ supplemented with 60 mg l^−1^ uracil if needed. Three independent single colonies, with relevant genetic modifications, were inoculated into 15 ml tubes with 2 ml of fresh minimal medium. Cultures were inoculated, from 24 h precultures, at initial OD_600_ = 0.05 in 15 ml of minimal medium and cultivated at 200 r.p.m. and 30 °C for 72 h. When shake-flask fermentations were conducted to mimic fed-batch conditions, Glucose FeedBeads (no. SMFB63361, Kuhner Shaker), corresponding to 30 g l^−1^ glucose, were used as the sole carbon source and cultivated for 96 h at 30 °C with 200 r.p.m. agitation.

### Fed-batch fermentation

Batch and fed-batch fermentations for FFA production were performed in 1.0 l bioreactors in a DasGip Parallel Bioreactors System (DasGip)^25^. The percentage of dodecane was maintained at almost 15% during the whole fermentation by the subsequent addition of further dodecane. Temperature, agitation, aeration and pH were monitored and controlled using a DasGip Control 4.0 System^36^. Aeration was initially provided at 36 standard litres per hour (sl h^−1^) and increased to a maximum of 48 sl h^−1^ depending on the level of dissolved oxygen. During fed-batch cultivation, cells were fed with a 400 g l^−1^ glucose solution at a rate that was exponentially increased (*m* = 0.05 h^−1^) to maintain a constant biomass-specific glucose consumption rate. The minimal medium contained 1.8 g l^−1^ (NH_4_)_2_SO_4_, 18 g l^−1^ KH_2_PO_4_, 3.0 g l^−1^ MgSO_4_ ∙ 7H_2_O, 360 mg l^−1^ uracil, 6× trace metal and 6× vitamin solution.

### Metabolite quantification

Free fatty acid titres in whole-cell culture (only FFAs were measured in this study) were quantified following previously published methods. Fatty acid extraction from the cell-free aqueous supernatant and dodecane phase was done following previously published methods^36^. Extracellular glucose, glycerol, ethanol and organic acid concentrations were determined by high-performance liquid chromatography analysis.

### Statistics and reproducibility

At least two independent experiments, begun from the cultivation of three, were performed for each quantification. Statistical analysis was conducted using Student’s *t*-test (two-tailed, two-sample unequal variance, *P < 0.05, **P < 0.01, ***P < 0.001; sample size, *n* = 3).

### Reporting summary

Further information on research design is available in the Nature Research Reporting Summary linked to this article.

## Supplementary information

Reporting Summary

Supplementary Table 1 Codon-optimized genes used in this study.

Supplementary Table 2 Strains used in this study.

Supplementary Table 3 Primers used in this study.

Supplementary Table 4 Plasmids used in this study.

Supplementary Table 5 Reagents used in this study.

## Data Availability

The data that support the findings of this study are available within the article, Supplementary Information files and Source Data files linked to each figure.

## Source data

Source Data Fig. 1 Statistical Source Data.
Source Data Fig. 2 Statistical Source Data.
Source Data Fig. 3 Statistical Source Data.
Source Data Fig. 4 Statistical Source Data.
Source Data Extended Data Fig. 4 Statistical Source Data.
Source Data Extended Data Fig. 5 Statistical Source Data.
Source Data Extended Data Fig. 6 Statistical Source Data.
Source Data Extended Data Fig. 7 Statistical Source Data.
Source Data Extended Data Fig. 8 Statistical Source Data.
Source Data Extended Data Fig. 9 Statistical Source Data.
Source Data Extended Data Fig. 10 Statistical Source Data.

## Code availability

No custom code was used in this paper. Gas chromatography–mass spectrometry and liquid chromatography–mass spectrometry data analyses were performed using published software, as indicated in Methods.
