# Author Correction: Metabolic reconfiguration enables synthetic reductive metabolism in yeast

**DOI:** 10.1038/s42255-023-00749-3

**Published:** 2023-02-07

**Authors:** Tao Yu, Quanli Liu, Xiang Wang, Xiangjian Liu, Yun Chen, Jens Nielsen

**Affiliations:** 1 Department of Biology and Biological Engineering, Chalmers University of Technology, Gothenburg, Sweden; 2 Novo Nordisk Foundation Center for Biosustainability, Chalmers University of Technology, Gothenburg, Sweden; 3 Novo Nordisk Foundation Center for Biosustainability, Technical University of Denmark, Kongens Lyngby, Denmark; 4 Center for Synthetic Biochemistry, CAS Key Laboratory of Quantitative Engineering Biology, Shenzhen Institute of Synthetic Biology, Shenzhen Institutes of Advanced Technology, Chinese Academy of Sciences, Shenzhen, China; 5 BioInnovation Institute, Copenhagen, Denmark

**Keywords:** Metabolic engineering, Cellular microbiology, Cell biology, Biocatalysis, Metabolism

Correction to: *Nature Metabolism* 10.1038/s42255-022-00654-1. Published online 27 October 2022.

In the version of this article originally published, there was a presentation error in Fig. 3c where symbols in the rows for *GDH1,2* and *NED1,2* appeared as plus rather than minus signs, as they now appear in the corrected HTML and PDF versions of the article.

