# Supplementary material for: Metabolic reconfiguration enables synthetic reductive metabolism in yeast
Source: Nat Metab. 2022 Oct 27;4(11):1551–9. doi: 10.1038/s42255-022-00654-1 (PMC9684072; doi:10.1038/s42255-022-00654-1)
Supplement: Supplementary file 1 — Reporting Summary [file 42255_2022_654_MOESM1_ESM.pdf]

nature portfolio

Corresponding author(s): Jens Nielsen

Last updated by author(s): Aug 18, 2022

Reporting Summary

Nature Portfolio wishes to improve the reproducibility of the work that we publish. This form provides structure for consistency and transparency in reporting. For further information on Nature Portfolio policies, see our Editorial Policies and the Editorial Policy Checklist.

Statistics

For all statistical analyses, confirm that the following items are present in the figure legend, table legend, main text, or Methods section.

n/a	Confirmed	
☐	☒	The exact sample size (n) for each experimental group/condition, given as a discrete number and unit of measurement
☐	☒	A statement on whether measurements were taken from distinct samples or whether the same sample was measured repeatedly
☐	☒	The statistical test(s) used AND whether they are one- or two-sided<br>*Only common tests should be described solely by name; describe more complex techniques in the Methods section.*
☒	☐	A description of all covariates tested
☒	☐	A description of any assumptions or corrections, such as tests of normality and adjustment for multiple comparisons
☐	☒	A full description of the statistical parameters including central tendency (e.g. means) or other basic estimates (e.g. regression coefficient) AND variation (e.g. standard deviation) or associated estimates of uncertainty (e.g. confidence intervals)
☐	☒	For null hypothesis testing, the test statistic (e.g. F, t, r) with confidence intervals, effect sizes, degrees of freedom and P value noted<br>*Give P values as exact values whenever suitable.*
☒	☐	For Bayesian analysis, information on the choice of priors and Markov chain Monte Carlo settings
☒	☐	For hierarchical and complex designs, identification of the appropriate level for tests and full reporting of outcomes
☒	☐	Estimates of effect sizes (e.g. Cohen's d, Pearson's r), indicating how they were calculated

Our web collection on statistics for biologists contains articles on many of the points above.

Software and code

Policy information about availability of computer code

Data collection	Xcalibur software; Agilent 1260-perfoemance liquid Chromatography; Gas Chromatography (GC, ThermoFisher Scientific); Gas Chromatography-Mass Spectrometer(GC-MS, ThermoFisher Scientific) and a DSQII mass 440 spectrometer;
Data analysis	Microsoft Excel for data analysis; Chromeleon 7 for GC analysis; Xcalibur(TraceFinder 3.3 General Quan) instrument for GC-MS analysis; Openlab CDS (LC1260 Infinity II) for HPLC analysis;

For manuscripts utilizing custom algorithms or software that are central to the research but not yet described in published literature, software must be made available to editors and reviewers. We strongly encourage code deposition in a community repository (e.g. GitHub). See the Nature Portfolio guidelines for submitting code & software for further information.

Data

Policy information about availability of data

All manuscripts must include a data availability statement. This statement should provide the following information, where applicable:

- Accession codes, unique identifiers, or web links for publicly available datasets
- A description of any restrictions on data availability
- For clinical datasets or third party data, please ensure that the statement adheres to our policy

The data that support the findings of this study are available from the corresponding author upon reasonable request. All plasmids and strains used in this study are available from the corresponding author under a material transfer agreement.

Field-specific reporting

Please select the one below that is the best fit for your research. If you are not sure, read the appropriate sections before making your selection.

- [x] Life sciences
- [ ] Behavioural & social sciences
- [ ] Ecological, evolutionary & environmental sciences

For a reference copy of the document with all sections, see nature.com/documents/nr-reporting-summary-flat.pdf

Life sciences study design

All studies must disclose on these points even when the disclosure is negative.

Sample size	Generally, three independent samples were adopted for data analysis due to relatively small deviation of microbial cells, which is also standard in metabolic engineering field that is sufficient. For some special experiments, more, or fewer sample sizes are adopted, as stated in the figure legends.
Data exclusions	No data were excluded from the analyses
Replication	All the biochemical and biological experiments were performed in three replications or more. For some specific experiments with huge data, or unsuccessful attempts, at least two replications were involved to draw conclusions, as illustrated and stated in the manuscript.
Randomization	This is not relevant to our study. Generally, the cultivation of microbial cells dose not require allocated experimental groups. In our study, yeast colonies from transformations were randomly selected for further analysis.
Blinding	This is not relevant to our study. Our samples are randomly picked colonies. For all experiments, the researchers were blind to the samples. All data were analyzed and checked by multiple authors and reviewed by the corresponding author.

Reporting for specific materials, systems and methods

We require information from authors about some types of materials, experimental systems and methods used in many studies. Here, indicate whether each material, system or method listed is relevant to your study. If you are not sure if a list item applies to your research, read the appropriate section before selecting a response.

Materials & experimental systems

n/a	Involved in the study	
☒	☐	Antibodies
☒	☐	Eukaryotic cell lines
☒	☐	Palaeontology and archaeology
☒	☐	Animals and other organisms
☒	☐	Human research participants
☒	☐	Clinical data
☒	☐	Dual use research of concern

Methods

n/a	Involved in the study	
☒	☐	ChIP-seq
☒	☐	Flow cytometry
☒	☐	MRI-based neuroimaging
